# Perspectives and challenges to discovering hemoglobin-inducing agents in Sickle Cell Disease

**DOI:** 10.3389/fmed.2022.1002063

**Published:** 2022-09-08

**Authors:** Aline Renata Pavan, Juliana Romano Lopes, Carlos Henrique Lima Imperador, Chung Man Chin, Jean Leandro dos Santos

**Affiliations:** ^1^Department of Drugs and Medicines, School of Pharmaceutical Sciences, São Paulo State University (UNESP), São Paulo, Brazil; ^2^Institute of Chemistry, São Paulo State University (UNESP), São Paulo, Brazil; ^3^Advanced Research Center in Medicine (CEPAM), School of Medicine, Union of the Colleges of the Great Lakes (UNILAGO), Sao Jose do Rio Preto, SP, Brazil

**Keywords:** Sickle Cell Disease, fetal hemoglobin, fetal hemoglobin inducers, epigenetic, NO/sCG/cGMP pathway, clinical trials, Cd34 blood cells+

## Introduction

Sickle Cell Disease (SCD), a distinct group of β-hemoglobinopathies, includes Sickle Cell Anemia (SCA) and β-thalassemia (1). An estimate of about 300,000 newborns was diagnosed with SCA worldwide, mainly in low-income countries such as sub-Saharan Africa, which contributed to about 75% of this statistic. SCD, a point mutation in the sixth codon of the β-globin gene (GAG to GTG), led to the replacement of glutamic acid by valine in the adult hemoglobin (HbA), thus, forming HbS, which in the deoxygenated state, prone to polymerization, modified the erythrocyte cytoskeleton into the well-known sickle shaped-form. These cells were susceptible to hemolysis after continuous oxy-deoxy cycles, contributing to chronic inflammation and nitric oxide depletion, which would worsen the vascular damage and cause the vaso-occlusion process (1–5).

Inflammation and vaso-occlusion, associated with multisystemic damage, were responsible for the clinical manifestations, including cardiovascular and pulmonary diseases, retinopathy, stroke, pain, acute chest syndrome, nephropathy, and priapism, among others. The diversity of symptoms was associated with the β-globin haplotypes among SCA patients (2). For example, fetal hemoglobin levels (HbF) could range from 0.1 to 30%, and those with SCA phenotypes exhibiting HbF persistence might have minor or lack symptoms (3). The polymorphism in genes associated with the pathophysiology of SCD, those involved in the chronic inflammatory process and vascular endothelial dysfunction, were responsible for the various clinical manifestations. The current research aimed to reduce the disease burden through symptom management to increase the expectancy and quality of life, which is a serious concern, mainly for developing countries, where the child mortality rate could go up to 90% before the age of five (4). For developed countries, such as the US, a reduction of up to 30 years in life expectancy was found by comparing SCD patients with healthy individuals, which seems inconceivable given the scientific progress in recent years (5).

Allogeneic hematopoietic stem cell transplant (allo-HSCT), the curative approach for SCA, could be applied to a small number of patients since about only 20% of them have a healthy HLA-identical sibling donor (6). In addition, the high cost, which was valued in the US estimate at $406,193, chronic graft vs. host disease, and high rates of morbidities made such an approach difficult in the clinical routine (7). Genetically modified autologous stem cells were an alternative for curing that included correcting the mutation associated with the disease through gene editing (i.e., CRISPR-Cas9), restoring HbF production by knockout transcription factors such as BCL11A, or including modified β-globin genes that avoid hemoglobin polymerization (i.e., LentiGlobin BB305) (7, 8). The cost for gene therapy was estimated to be above 1 million USD (6), and access to this strategy was limited, but in the long run, gene therapy could be a safer alternative than allo-HSCT.

Clinical care included focusing on hydration, immunization, blood transfusion, and pain management (9). The most difficult part of SCA care was the limited number of approved drugs to reduce and prevent the symptoms, which included only four drugs, approved by the US FDA: hydroxyurea (HU), L-glutamine, crizanlizumab, and voxelotor (10). Under the preclinical perspective, the drug discovery was focused on preventing HbS polymerization, vascular adhesion, and coagulation; reducing the inflammatory process, oxidative stress, and nitric oxide/sCG/cGMP pathway impacts; and promoting HbF induction (11, 12). HU promoted the HbF induction, a validated approach, for which it acted through pleiotropic effects, including the activation of the enzyme sGC, which increases the level of cGMP; and the downregulation of the silencing transcription factors BCL11A, KLF-1, and MYB (13, 14). The regulation of the expression of the gamma-globin gene was the molecular basis for HbF induction. Medicinal Chemistry approaches were used to design new compounds to inhibit epigenetic enzymes, and transcription factors, act directly on the NO/sCG/cGMP pathway and induce HbF production.

**Figure 1:**
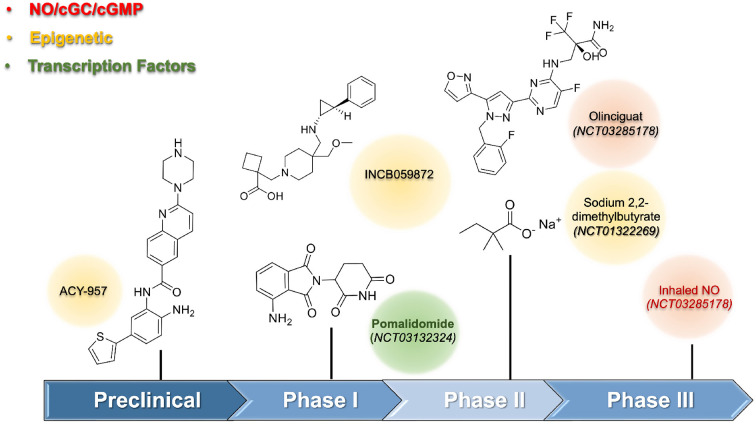
Emerging HbF inducers for Sickle Cell Disease.

During the preclinical stage, the identification of HbF inducer used phenotypic assays culture cells (i.e., K562; CD34+, HUDEP-2 cells). The preliminary results obtained from screening were validated by a secondary assay using different cell lines since the use of human CD34+ progenitor cells were mandatory to reduce false-positive results. *In vivo* assays using transgenic animals were performed to confirm the efficacy of the HbF-inducing agent. One of the first HbF-inducing agents, except for HU, investigated, was the short-chain fatty acids. The preclinical data suggested its potential as a new drug; however, the irregular and poor pharmacokinetics limited its use in humans. Moreover, the clinical trials of sodium 2,2-dimethylbutyrate revealed a limited effect by increasing at 2% the levels of HbF, and only 2.7% when combined with HU (NCT01322269) (15). Molecular studies for short-chain fatty acids induced HbF through inhibition of histone deacetylase (HDAC) enzymes. HDAC, an epigenetic enzyme constituted of eighteen HDAC isoforms distributed in four classes: I (HDAC-1, 2, 3, and 8), IIA (HDAC-4, 5, 7, and 9), and IIB (HDAC-6 and 10), III (sirtuins 1–7), and IV (HDAC-11). HDAC, acted by removing the acetyl group from ε-N-acetyl-lysine in histone tails, and thus, regulated gene transcription of the γ-globin gene, whose expression provided selective inhibition of HDAC-1 and HDAC-2 (16). Structural requirements to design selective class I, specifically HDAC-1 and HDAC-2, were described elsewhere (17). Compound ACY-957, a 2-aminobenzamide derivative, selectively inhibited HDAC-1 and HDAC-2 with IC50 values of 7 nM and 18 nM, respectively, and showed a favorable pharmacokinetic profile in mammals and rodents in preclinical studies. For example, monkeys treated with ACY-957 (25 and 75 mg/Kg) increased HbF levels, but white blood suppression observed during the treatment disappeared after washout time. Alternative schemes considering non-daily administration were well tolerated (Figure 1).

The inhibition of epigenetic enzymes was pursued, including lysine-demethylase 1 (LSD-1), which had pronounced effects on HbF induction. *In vitro* and *in vivo* studies using transgenic sickle mice validated LSD-1 as a promising target as a HbF-inducing agent (18, 19). A phase I study (US Clinical trial: NCT03132324) was initiated using the LSD-1 inhibitor named INCB059872 but was terminated due to a business decision. Pharmacological interventions were important to regulate γ-globin gene expression, and their interference with several transcription factors, including TR4, BCL11A, KLF-1, MYB, SOX-6, GATA-1, Nrf2, and FOXO3 had been associated with HbF production. However, their involvement was a vital process, and low druggability caused serious concerns about long-term safety in the use of compounds interfering with transcription factors. The drug pomalidomide, which modulates the levels of SOX-6, here, held importance. The additional anti-inflammatory effects, beyond its interference, helped in SCA treatment. Phase I trials using pomalidomide at 4 mg/day for 12 weeks reported a significant increase in the HbF levels (Clinical Trial: NCT01522547) (20).

Guanylate cyclase and the NO/cGMP signaling pathway presented another promising approach to finding out new HbF-inducing agents (Figure 1). The involvement of the soluble guanylate cyclase (sGC) in physiological processes such as vasodilation, platelet, and leukocyte adhesion had beneficial pleiotropic effects beyond HbF induction. Olinciguat, an sGC stimulator able to induce γ-globin mRNA expression up to 2.9-fold at 10 μM, received orphan drug status from the US FDA in 2018 to treat SCA. This drug showed both anti-inflammatory effects and prevented vaso-occlusion events (21, 22). A phase-II trial (Clinical Trial: NCT03285178) was terminated, revealing that the drug was safe and well tolerated by SCA patients. As NO levels were reduced in SCA patients, its reestablishment could improve vascular homeostasis. The beneficial effects of NO-donors to increase HbF levels are accompanied by concerns regarding its adverse cardiovascular effects. Thus, the kinetic effects of NO-release as well as the use of appropriate formulations need further investigation. A phase III trial with inhaled NO (Clinical Trial: NCT03285178) did not show a reduction in the time to solve the vaso-occlusive crisis, although other outcomes improved (23).

The current status of some clinical trials found in the US clinical trials database (https://clinicaltrials.gov/) involving HbF-inducing agents was still limited. Some of these studies are investigating the use of HU in pediatric patients (NCT01506544; NCT00305175) or in combination with other drugs (i.e., crizanlizumab - NCT03814746; tadalafil - NCT05142254; and clotrimazole - NCT00004492). It has been estimated that the rate of HU failure is about 30%, however, patients-related issues such as the lack of treatment adherence, adverse-effects, failures to health access and medicines, and a non-optimal dose schemes could be the main reasons to contribute for so high levels of failure, suggesting that rate of non-responsiveness must be lower. The investigations on safe and efficacious new drugs acting as HbF-inducing agents are valuable and could represent an alternative for HU. Despite the expectancy toward the gene editing technologies regarding the cure of genetic diseases, the implementation of this approach into the clinic is expensive and demands medical facilities, highly specialized workers, and involves high risks for patients. Considering the diversity of SCD symptoms, the use of HbF-inducing drugs will represent an alternative for many patients, mainly in low-income countries for the next few years. An efficacious treatment was to take into account interventions due to the multifactorial aspects of SCA and the diversity of phenotypes through various pathways to control the main symptoms in many aspects. There were a lot of perspectives regarding the future of HbF-inducers, and the authors believed that additional efforts to investigate the drug discovery of polypharmacology drugs could provide a promising start. Even after advancements in gene therapy, the use of small molecules would be an important part of the treatment, considering that the diversity of clinical manifestations and the NO/sCG/cGMP pathway to be a promising approach for drug discovery in the future.

## Author contributions

Conceptualization and writing—original draft preparation: AP, JL, CL, JS, and CM. Supervision, project administration, and funding acquisition: JS and CM. All authors have read and agreed to the published version of the manuscript.

## Funding

AP was supported by the School of Medicine, the Union of the Colleges of the Great Lakes (UNILAGO). SJRP, SP, and the Program for the Scientific Development (PADC-UNILAGO), Brazil. Fundação de Amparo à Pesquisa do Estado de São Paulo (FAPESP) process numbers 21/100059-9, 20/13279-7, 19/10746-6, and 18/19523-7.

## Conflict of interest

The authors declare that the research was conducted in the absence of any commercial or financial relationships that could be construed as a potential conflict of interest.

## Publisher's note

All claims expressed in this article are solely those of the authors and do not necessarily represent those of their affiliated organizations, or those of the publisher, the editors and the reviewers. Any product that may be evaluated in this article, or claim that may be made by its manufacturer, is not guaranteed or endorsed by the publisher.
